# Whole genome methylation analyses of schizophrenia patients before and after treatment

**DOI:** 10.1080/13102818.2014.933501

**Published:** 2014-08-26

**Authors:** Blaga Rukova, Rada Staneva, Savina Hadjidekova, Georgi Stamenov, Vihra Milanova, Draga Toncheva

**Affiliations:** ^a^ Department of Medical Genetics, Medical University of Sofia, Sofia, Bulgaria; ^b^ Women's Health Hospital “Nadezhda”, Sofia, Bulgaria; ^c^ Department of Psychiatry, Medical University of Sofia, Sofia, Bulgaria

**Keywords:** DNA methylation, whole genome arrays, schizophrenia, treatment

## Abstract

The aetiology of schizophrenia is still unknown but it involves both heritable and non-heritable factors. DNA methylation is an inheritable epigenetic modification that stably alters gene expression. It takes part in the regulation of neurodevelopment and may be a contributing factor to the pathogenesis of brain diseases. It was found that many of the antipsychotic drugs may lead to epigenetic modifications. We have performed 42 high-resolution genome-wide methylation array analyses to determine the methylation status of 27,627 CpG islands. Differentially methylated regions were studied with samples from 20 Bulgarian individuals divided in four groups according to their gender (12 males/8 females) and their treatment response (6 in complete/14 in incomplete remission). They were compared to two age and sex matched control pools (110 females in female pool/110 males in male pool) before and after treatment. We found significant differences in the methylation profiles between male schizophrenia patients with complete remission and control male pool before treatment (*C16orf70*, *CST3*, *DDRGK1*, *FA2H*, *FLJ30058*, *MFSD2B*, *RFX4*, *UBE2J1*, *ZNF311*) and male schizophrenia patients with complete remission and control male pool after treatment (*AP1S3*, *C16orf59*, *KCNK15*, *LOC146336*, *MGC16384*, *XRN2*) that potentially could be used as target genes for new therapeutic strategies as well as markers for good treatment response. Our data revealed major differences in methylation profiles between male schizophrenia patients in complete remission before and after treatment and healthy controls which supports the hypothesis that antipsychotic drugs may play a role in epigenetic modifications.

## Introduction

Schizophrenia is a severe debilitating disorder affecting approximately 1% of the population worldwide. The aetiology of schizophrenia is still unknown but it involves both heritable and non-heritable factors.[1] Symptoms are divided into three main groups: positive, negative and cognitive. Most common positive symptoms are hallucinations, delusions, thought and movement disorders. Most often they respond well to therapy. Negative symptoms are associated with deregulation of emotions, behaviour and thoughts. They are non-specific and can be easily associated with other disorders, thus making correct diagnosis of schizophrenia more difficult. Negative symptoms such as apathy, abulia, anhedonia and poverty of speech do not respond so well to treatment. The third group includes cognitive dysfunction – impaired functioning of memory and overall disorganization.[2–4] Schizophrenia is a serious social and economic burden for the patients, their families and the society. Treatment with antipsychotic drugs does not cure but only relieves schizophrenia symptoms. Elucidation of the schizophrenia etiological mechanisms is of great importance for the development of novel therapeutics and better treatment.

Epigenetics includes heritable dynamically regulated changes responsible for regulation of gene expression that do not affect the primary DNA sequence.[5] Epigenetic modifications are involved in normal organism development and in particular in embryonic and postnatal nervous system development and cerebral function. It plays a role in neurogenesis, development, differentiation and neuroplasticity.[6] There is data in support of the thesis that aberrant epigenetic changes participate in vulnerability to psychiatric disorders.[7–9]

Epigenetic characteristics can be modulated throughout life: from embryonic phase to adulthood. Epigenetic remodelling is influenced by environmental factors such as nutrition, drugs, physical and psychosocial factors.[10] Epigenetic modifications are reversible and susceptible to environmental influences so they may prove to be very suitable target for the discovery of new potential drug treatment strategies.

One of the most important epigenetic modifications is DNA methylation. It takes part in the regulation of neurodevelopment and may be involved in pathogenesis of brain diseases.[11,12] There is literature data about involvement of some psychiatric drugs in epigenetic regulation. The aim of our study is to prove the presence of methylation profile differences before and after treatment with currently approved treatment protocols thus laying the basis for future research on drugs affecting epigenetic characteristics as potential new treatment strategy.

Still the effect of antipsychotic drugs on epigenetic modifications is not well established. A study of global DNA methylation in leukocytes of patients with schizophrenia discovered significant global hypomethylation, while haloperidol treatment resulted in an increase in methylation.[13]

It was found that many of the antipsychotic drugs may lead to epigenetic modifications. Valproic acid inhibits histone deacetylases and leads to DNA demethylation. Haloperidol also causes histone modifications and global DNA demethylation in female rat brains, although there is evidence that haloperidol treatment is unrelated to the methylation status of the reelin and *GAD67* genes in the frontal cortex or striatum.[14,15]

Clozapine treatment increases H3K4 trimethylation of *GAD67* gene in prefrontal human cortex. Clozapine decreases methionine-induced hypermethylation of reelin promoter and increases histone acetylation. Also it induces histone hyperacetylation.[16,17]

In the frontal cortex and striatum of mice after treatment with sulpiride and amisulpiride it is observed an increased histone acetylation and decreased methylation level in the reelin gene promoter.[17] Risperidone supports striatum global H3 phospho-acetylation.[18]

There are data suggesting the involvement of clozapine, quetiapine and olanzapine in histone modifications and for decrease of DNA methylation in GABAergic or glutamatergic neurons.

Other studies present quite controversial results about haloperidol treatment and its effects on epigenetic profiles. Some experiments showed that it did not influence histone modifications, DNA methylation and there was no evidence of change in the histone acetylation pattern in striatopallidal neurons, while there was an increase of histone phosphorylation.[17–19]

These data demonstrate involvement of typical or atypical antipsychotic drugs in epigenetic remodelling. In our study we screened almost all CpG islands found so far in the same patient before and after treatment. We aimed to discover new genes with differentially methylated regions (DMRs) that could potentially be used as a target for new antipsychotic drugs. We hope to define possible epigenetic markers as an additional tool for objective evaluation of the therapeutic effect.

## Materials and methods

We have examined the methylation status of 20 individual Bulgarian patients with schizophrenia (8 females and 12 males) before and after treatment, and compared them to pools of sex and age matched healthy controls. Informed consent was obtained from all investigated subjects and the relevant Ethics Committees of the hospitals where subjects were recruited gave approval for the use of these samples in genetic studies. The diagnosis of schizophrenia was made by experienced psychiatrists, according to Diagnostic and Statistical Manual of Mental Disorders, 4th Edition (DSM-IV) criteria on the basis of extensive clinical interviews.

Methylation analysis was performed using the Agilent Human DNA Methylation Microarray platform with density of 237,227 sequences covering 27,627 CpG islands (1 × 244K). All included arrays passed standard quality control metrics.

We extracted gDNA from peripheral blood by standard phenol–chloroform extraction technique.[20] Concentration and purity of the gDNA were determined with NanoDrop 2000C (Thermo scientific). All samples were electrophoretically tested for verification of the gDNA integrity. We used methylated DNA immunoprecipitation (MeDIP) protocol for extraction of the methylated DNA fraction. It uses a monoclonal antibody against 5-methylcytosine to enrich for the methylated fraction of a genomic DNA sample.[21,22] Separated methylated DNA (sample) and whole genome DNA (control) from the same patient were labelled according to the instructions of the manufacturer and then were hybridized on the array.

Agilent methylation microarrays were scanned, using Agilent High-Resolution Microarray Scanner G2505 with resolution of 2 μm. Scans were performed at a wavelength of 532 nm for green laser and 635 nm for red laser. The resulting .tif images were processed with Agilent Feature Extraction software v.11.0.1.1 and Agilent Workbench 6.5.0.18, according to the manufacturer's instructions.

A unified specific algorithm for analysis of results is not available as such studies are still novel. The most suitable algorithm for the MeDIP methylation data analysis is considered BATMAN criterion. Bayesian tool for methylation analysis (BATMAN) enables the estimation of absolute methylation levels from immunoprecipitation-based DNA methylation profiles. This parameter can have the following values: −1 (hypomethylation); 1 (hypermethylation); or 0 (uninterpretable).[23] We designed special software to help interpretation of 244,000 positions for each array. This software estimates the methylation status of the whole CpG island and compares island methylation status across the arrays in order to search for DMRs. The methylation status is determined as a percentage of methylated probes in the island. The results with all DMRs for one patient array, compared to healthy control array, are presented as a separate excel table. We excluded genes with uninterpretable status. According to the literature when over 60% of a CpG island is methylated it is defined as ‘methylated’ and if below 40% of CpG island is methylated it is considered as ‘unmethylated’.[23] CpG islands with methylation status in the range 40%–60% are considered as ‘intermediate methylated’ and are not addressed in our study.[23]

## Results and discussion

The methylation status of 20 patients with schizophrenia (8 females and 12 males) was analysed by us on 40 microarrays as separate slides were used before and after treatment. We compared the methylation profile of patients to age and sex matched pools of healthy controls. According to our criteria for data analysis we compared the number of DMRs before and after treatment of the same patient to healthy control pool of the same sex (110 healthy female controls and 110 healthy male controls in each pool, respectively). We split patients according to gender and effect of treatment into four groups:

1. Females with complete remission: three patients;
2. Females with incomplete remission: five patients;
3. Males with complete remission: three patients;
4. Males with incomplete remission: nine patients.

The results from this analysis are shown in Tables 1 and 2.

**Table 1.** Number of DMRs in the female groups.

Female patients compared to female pool of healthy controls								
	Complete remission			Incomplete remission				
Patient (no.)	1	2	3	4	5	6	7	8
DMRs before treatment (*n*)	608	357	749	1205	359	1184	885	255
DMRs after treatment (*n*)	1057	479	260	1155	902	1155	354	753

**Table 2.** Number of DMRs in the male groups.

Male patients compared to male pool of healthy controls												
	Complete remission			Incomplete remission								
Patient (no.)	1	2	3	4	5	6	7	8	9	10	11	12
DMRs before treatment (*n*)	534	467	455	496	726	1648	747	502	521	625	387	666
DMRs after treatment (*n*)	555	934	397	827	576	1252	628	754	537	487	390	1064

A reduction in the number of DMRs was observed in the results of some patients while the analysis for others showed just the opposite – an increase in the number of DMRs. There was also a third group of patients with nearly the same number of DMRs before and after treatment. These differences in the number of DMRs did not correlate with the treatment response.

The second step in data analysis was to compare the patients in each group according to their DMRs. The used criterion for significant DMRs was their presence in more than 50% of patients in the respective group. DMRs which occurred in all patients were defined by us to be the most important and they may be considered as disease-associated (Table 3).

**Table 3.** Number of DMRs that are present in >50% and 100% from the sets in the ‘before’ and ‘after’ treatment groups among the schizophrenia patients.

	Females				Males			
	Complete remission		Incomplete remission		Complete remission		Incomplete remission	
	DMRs before treatment (*n*)	DMRs after treatment (*n*)	DMRs before treatment (*n*)	DMRs after treatment (*n*)	DMRs before treatment (*n*)	DMRs after treatment (*n*)	DMRs before treatment (*n*)	DMRs after treatment (*n*)
>50% of the patients	867	785	627	680	306	293	299	291
100% of the patients	26	16	46	77	56	46	14	11

The last analysis that was performed by us defined the DMRs that occurred in 100% of the patients in the respective group (females in complete remission, males in complete remission, females in incomplete remission and males in incomplete remission) before and after treatment. Then we excluded the DMRs that were present in both sets (50% and 100%) in the ‘before’ and ‘after’ treatment groups since their methylation status remained unchanged. The DMRs that appeared to be unique for the ‘before treatment’ and ‘after treatment’ groups were considered of interest, since these represented a difference most likely caused by the treatment. The aim of this analysis was to check if there were genes that were related to the effect of treatment (the ones that are solely presented in the ‘after treatment’ group) and genes that could be potential targets for new therapies (the ones that are unique to the ‘before treatment’ group).

According to their treatment response the groups with patients in incomplete remission showed high diversity. It should be noted that the diagnosis is very subjective, because it is based on interviews. Therefore, the information from the interviews can be differently interpreted, leading to difficulties in the explanation and analysis of the data for the methylation profiles. All DMRs that were determined before treatment in all patients from the female group in incomplete remission reoccurred after treatment in 50% and more of the patients. Only a single DMR (*EXOSC6*) out of 77 that was not found before treatment was determined in the same group after treatment. All patients in this group had a hypermethylated CpG island (CpG: 124) inside the gene body of *EXOSC6*. Yet this data cannot be interpreted unambiguously because the effect of methylation in regions different than that of the promoter is still unclear.[24,25] The exact function of the product encoded by this gene is still unknown but it is assumed that it plays role in mRNA degradation.[26] However, any published data about its involvement in the pathogenesis of schizophrenia were not found.

Genes with DMRs that met the analysis criteria for significance were not found by us among the patients in the male group in incomplete remission.

It has been known that there is a difference between the methylation status of males and females with schizophrenia.[27] The disease among male patients is associated with earlier onset, increased hostility and worse overall premorbid functioning than in females.[28,29] One specific out of 26 common DMRs before treatment (*MIR181C*) and 1 out of 16 after treatment (*BCOR*) were found in the female group in complete remission. These genes are involved in transcription regulation and ubiquitination. However, as these DMRs are located in different CpG islands from different patients we cannot consider them credible and the data cannot be correctly interpreted (Table 4). This is attributed to the small number of patients in this group. Another difficulty, as already pointed out, refers to the diagnosis of schizophrenia, which may be subjective because it is based on an interview with the patient. It should also be taken into consideration that epigenetic modifications are sensible to environmental factors. Thus, the obtained methylation profile from blood samples can be affected by many other factors that are not related to schizophrenia and treatment effects.

**Table 4.** Function of genes with DMRs in female patients in complete remission.

Females in complete remission						
Gene name	Gene function	CytoBand	ChrName	Description	CpG	Methylation status
Before treatment						
*MIR181C*	Post-transcriptional regulation	p13.13	chr19	PROMOTER	117/41	Hypermethylated
After treatment						
*BCOR*	Transcriptional corepressor, protein ubiquitination	p11.4	chrX	INSIDE	54/75/168	Hypermethylated

The data analysis of the male group in complete remission presented more interesting results. Among the group, 9 specific out of 56 common DMRs before treatment (*C16orf70*, *CST3*, *DDRGK1*, *FA2H*, *FLJ30058*, *MFSD2B*, *RFX4*, *UBE2J1*, *ZNF311*) and 6 out of 46 after treatment (*AP1S3*, *C16orf59*, *KCNK15*, *LOC146336*, *MGC16384*, *XRN2*) were identified. All DMRs from these genes are located in the same region of the gene in all patients. Therefore, these can be accepted by us as reliable. It also shows the importance of good patient selection in this group from clinical perspective and could explain the results. It is proposed by us that these genes should be related to the pathogenesis and treatment effect in male schizophrenia patients. The genes with DMRs in the groups with complete remission and the functions of these genes are presented in Table 5. Some of these genes are involved in transcription regulation, sphingolipid metabolism and protein degradation, whereas the function of others is still unknown. However, it is known that hypermethylation of the gene promoters suppresses transcription while hypomethylation activates it. The transcriptional significance of the methylation status of CpG islands in other DNA regions cannot be accurately interpreted.[24] Four of the genes with DMRs that were found before the treatment were hypermethylated (two in the promoter region, one inside the gene body and one downstream of the promoter). Four genes were hypomethylated before the treatment with DMRs inside the gene body. Following the treatment five of the genes had hypermethylated CpG islands (two in the promoter, two downstream of the promoter and one inside the gene body) and one gene presented a hypomethyated CpG island inside the gene body. The function of four genes is still unknown and therefore, these can be proposed as potential new genes that are related to the aetiology of schizophrenia. The other identified genes are: *CST3*, *FA2H*, *RFX4*, *UBE2J1*, *ZNF311*.

**Table 5.** Function of genes with DMRs in male patients in complete remission.

Males in complete remission						
Gene name	Gene function	CytoBand	ChrName	Description	CpG	Methylation status
Before treatment						
*C16orf70*	Unknown function	q22.1	chr16	PROMOTER	CpG: 44	Hypermethylated
*CST3*	Inhibitor of cysteine proteinases	p11.21	chr20	INSIDE	CpG: 84	Hypomethylated
*DDRGK1*	Unknown function	p13	chr20	INSIDE	CpG: 47	Hypermethylated
*FA2H*	Sphingolipid metabolic process	q23.1	chr16	INSIDE	CpG: 78	Hypomethylated
*FLJ30058*	Unknown function	q26.1	chrX	INSIDE	CpG: 63	Hypomethylated
*MFSD2B*	Unknown function	p23.3	chr2	PROMOTER	CpG: 66	Hypomethylated
*RFX4*	Transcription factor	q23.3	chr12	PROMOTER	CpG: 41	Hypermethylated
*UBE2J1*	Ubiquitin-mediated proteolysis	q15	chr6	INSIDE	CpG: 103	Hypomethylated
*ZNF311*	Transcription factor	p22.1	chr6	DOWNSTREAM	CpG: 42	Hypermethylated
After treatment						
*AP1S3*	Intracellular protein transport	q36.1	chr2	INSIDE	CpG: 79	Hypermethylated
*C16orf59*	Unknown function	p13.3	chr16	PROMOTER	CpG: 67	Hypermethylated
*KCNK15*	Potassium channel protein	q13.12	chr20	PROMOTER	CpG: 70	Hypomethylated
*LOC146336*	Unknown function	p13.3	chr16	DOWNSTREAM	CpG: 22	Hypermethylated
*MGC16384*	Unknown function	q24.33	chr12	PROMOTER	CpG: 88	Hypermethylated
*XRN2*	RNA metabolism	p11.22	chr20	DOWNSTREAM	CpG: 53	Hypermethylated

*CST3* gene encodes an inhibitor of cysteine proteinases. Normally the product of this gene is found in biological fluids and especially in the cerebrospinal fluid that surrounds and protects the brain and spinal cord.[30] We suppose that this gene is probably inhibited because in our study before the treatment it is hypomethylated inside the gene body.[24] The gene's function is related to formation of amyloid plaques, thus it has already been shown to be involved in some brain disorders.[31] Nevertheless, data concerning any participation of this gene in the etiopathogenesis of schizophrenia are not present yet. Based on our results it may be suggested that the gene could be considered to be important for the disease development.

*FA2H* catalyses the synthesis of 2-hydroxysphingolipids. There are reports in the literature for mutations in this gene that are involved in the development of neurodegenerative diseases but not specifically related to schizophrenia.[32,33] It is known that it is expressed in brain cells.[33] The result of our study shows that this gene has a hypomethylated DMR inside its gene promoter, which makes us believe that the gene is in inactivated state. Moreover, we suppose that *FA2H* can play a role in the etiopathogenesis of schizophrenia.

*RFX4* and *ZNF311* encode transcription factors. *RFX4* is associated with brain gliomas. Some of its isoforms are brain-specific while others are expressed in gliomas.[34,35] The DMR of this gene according to the study results is in a hypermethylated state in its promoter region, so it is deactivated, which could potentially contribute to development of abnormalities in basic brain processes. Dysregulation in the expression pattern of this gene can be associated with development of schizophrenia.

*UBE2J1* takes part in ubiquitination processes.[36] As a result of our study we found a hypomethylated DMR inside the gene body. We speculate that this gene is inactivated, leading to improper ubiquitination processes and subsequently to a putative mechanism for development of schizophrenia.

As a result of our experiments we report nine genes with different methylation profiles that are potential new candidate genes for novel drug therapy. The affected genes are involved in several major processes such as protein degradation, transcription regulation and sphingolipid metabolism.

The results after the treatment of the studied patients showed that these genes restored their methylation status. Also, another six genes changed their methylation profiles. All these may be attributed to the effect from the treatment and compensatory mechanisms in the organism. The methylation status of DMRs following the treatment was different compared to the results before it with five out of six genes being hypermethylated and one hypomethyalted. These genes are: *AP1S3*, *C16orf59*, *KCNK15*, *LOC146336*, *MGC16384*, *XRN2*.

*C16orf59*, *LOC146336*, *MGC16384* are not yet thoroughly studied and currently their function remains unknown. *AP1S3* is involved in protein sorting and intracellular transport.[37] The influence of drug treatment on its expression has not been reported so far and it could be a new marker for evaluation of treatment effect.

*KCNK15* encodes a potassium channel.[38] Our study shows that this gene appears to be activated since its promoter DMR is hypomethylated. The gene is expressed in the central auditory nervous system. We suggest that activation of the gene may be a result from the antipsychotic treatment, leading to compensation of some of the schizophrenic symptoms. This gene should also be considered to be used as a new candidate marker with potential to evaluate the effect from the therapeutic regimen. Eventually, these data could be applied in further studies: such as aiming to identify new drug targets and elucidate the disease pathology.[39]

*XRN2* gene encodes 5′-3′ exoribonuclease 2. It plays a role in RNA metabolism.[40] As far as our knowledge, data concerning the effect of antipsychotic drugs on its expression have not been published. The analysis of the results shows that the identified DMR is hypermethylated downstream of the promoter and we speculate that this could activate the transcription of the gene.

The current study reports difference in the methylation profiles of six genes that could be associated with the positive effect from the applied treatment of schizophrenia. One gene is known to encode a potassium channel. The function of some others is still unknown or unclear. It is believed that several processes such as intracellular transport and RNA metabolism are affected. Because the effect from the treatment on the expression of these genes has not been studied until now, it may be worth to thoroughly investigate the described genes in future studies addressing their possible role as new markers for treatment response.

## Conclusions

A whole genome methylation analysis on individual schizophrenia patient samples before and after treatment was performed in our study. As a result we found gender-specific differences in the methylation profile of patients that may be related to the etiopathogenesis of schizophrenia as well as to the effect from the applied treatment. Some of these differences could be potentially studied further as new therapeutic targets.

The results from our investigation were shown statistically significant only for the male patient group in complete remission. This points out that epigenetic modification by DNA methylation is more important for male patients compared to females. We suppose that several new genes could be potential targets for new drug discovery: *C16orf70*, *CST3*, *DDRGK1*, *FA2H*, *FLJ30058*, *MFSD2B*, *RFX4*, *UBE2J1* and *ZNF311*. Some of these take part in transcription regulation, ubiquitination and sphingolipid metabolism. The analysis of the ‘after treatment’ data revealed several genes as potential markers for good treatment effect: *AP1S3*, *C16orf59*, *KCNK15*, *LOC146336*, *MGC16384* and *XRN2*. They are involved in RNA metabolism, protein transport and sorting, and one is a potassium channel.

Nevertheless, the current study is still preliminary and we believe that more in-depth studies in larger cohorts of patients are required.
